# Auditory Discrimination Between Function Words in Children and Adults: A Mismatch Negativity Study

**DOI:** 10.3389/fpsyg.2015.01930

**Published:** 2015-12-22

**Authors:** Anna Strotseva-Feinschmidt, Katrin Cunitz, Angela D. Friederici, Thomas C. Gunter

**Affiliations:** Department of Neuropsychology, Max Planck Institute for Human Cognitive and Brain SciencesLeipzig, Germany

**Keywords:** mismatch negativity, children, word frequency, sentence comprehension, auditory

## Abstract

Previous behavioral studies showed that it is not until around the age of seven that German children reliably use case markers for the interpretation of complex sentences. Some explanations of this late development suggested that children might have difficulties in perceptual differentiation between function words that carry case information. We tested this hypothesis by using the neurophysiological index of pre-attentive discrimination, the mismatch negativity (MMN). Our data showed that children at the age of 3 years are able to automatically discriminate between the two determiner forms *der* and *den* when presented out of sentential context. The determiner form *der* elicited a more mature MMN response in children than the form *den*. In adults, the MMN pattern also differed with *der* showing an earlier peak than *den*. These findings indicate that *der* is easier to process than *den*, which in turn is related to the occurrence frequency of the determiner forms in language.

## Introduction

At the age of about 2 years, children start to produce their first two- and three-word combinations (Guasti, 2002; Szagun, 2006). In the following period, a rapid development in the acquisition of syntax takes place. One of the fundamental challenges of this development is the ability to detect and interpret linguistic features that encode thematic relations between participants of an utterance. The roles of sentence participants, that is, who is the agent and who is the patient of the action, can be expressed by different means. In German, this function is essentially held by morphological coding of case (MacWhinney et al., 1984; Kempe and MacWhinney, 1999). Nominative case forms usually mark agents, while accusative forms often mark patients in active declarative sentences. For example, in a less frequent but grammatically correct object-first structure *Den*_*acc*_ *Radfahrer beobachtet der*_*nom*_ *Polizist*. “The policeman watches the cyclist.,” the determiner forms indicate that the policeman is an actor, and the cyclist is the patient. However, children do not completely rely on morphological information when they interpret such sentences until they reach the age of 5-7 years (Schaner-Wolles, 1989; Primus and Lindner, 1994; Knoll et al., 2012). For example, in the study by Dittmar et al. (2008), which examined the morphological strategy in groups of children with mean ages of 2 years 7 months (hereafter ages are reported in the format [years;months]), 4;10, and 7;3, only the oldest group performed above chance level when exposed to complex sentences with unambiguous case-marking. Schipke et al. (2012) presented 3;0-, 4;6-, and 6;0-year olds with object-first sentences of type *Den*_*acc*_ *Frosch küsst der*_*nom*_ *Tiger* “The tiger kisses the frog” in a picture-matching task. None of the age groups responded systematically well to such sentences. The question arises as to why young language learners do not use the highly reliable accusative case marker in the course of their early linguistic development.

One of the explanations suggests that purely perceptual constraints have an effect on the acquisition of case. For example, MacWhinney et al. (1985) who investigated the processing of case-marking in Hungarian children of 2;6–6;0 years, suggested that the late reliance on case markers can be explained by their poor detectability. In Hungarian, the nominative and accusative case forms may only differ in the final consonant, for example, *squirrel*_*nom*_ [mocus] vs. *squirrel*_*acc*_ [mocust]. In the analysis of case-marking in the speech of German-acquiring children with normal hearing or with cochlear implants, Szagun (2004) similarly pointed to the lack of perceptual salience for the articles that carry case and gender information in German. Difficulties in the acquisition of the case system were explained by the missing accenting in sentential context and by the low discriminability between some determiner forms, for example, *den*_*acc*_ and *dem*_*dat*_. Furthermore, Szagun (2004) demonstrated that for some forms, children's construction of case categories interacted with the input frequency of the articles obtained from the child-directed speech of adults. The analyses indicated that children, instead of the accusative form *einen*_*acc*_, often used the highly frequent nominative form of the indefinite article *ein*_*nom*_, erroneously. In other words, children tended to make use of the form that occurred more frequently in adult speech.

The present study aims to assess the issues of perceptual discriminability and frequency of occurrence from the neurophysiological perspective. Firstly, we addressed the question of auditory discrimination between determiners. Secondly, we evaluated the impact of occurrence frequency on the discriminative abilities of 3-year-olds and adults. The study examined two forms of German definite articles that were previously investigated in sentential context (cf. Schipke et al., 2012), namely the nominative masculine singular form *der* and the accusative masculine singular form *den*. To assess children's discriminative abilities, we used an electrophysiological marker of automatic change detection *the mismatch negativity* (MMN; Näätänen, 1995). MMN is a negative deflection of the difference wave that is obtained by subtracting the brain response elicited by a *standard* (frequently presented) sound from a *deviant* (infrequently presented) sound in a so-called oddball paradigm. In adults, the MMN usually peaks between 100 and 250 ms after stimulus onset in response both to speech and non-speech deviants (for reviews, see Näätänen, 1995, 2001; Pulvermüller and Shtyrov, 2006). The latency of the MMN peak has been shown to be negatively correlated with age (Shafer et al., 2000, 2010; Morr et al., 2002). In 3-year-old children, the MMN peak has been observed between 120 and 400 ms post-deviance (Glass et al., 2008; Putkinen et al., 2012; Paquette et al., 2013). For example, the processing of vowel contrasts was associated with the MMN peak between 300 and 400 ms in Finnish 3-year-olds (Čeponienė et al., 2003). MMN to the contrast /ba - da/ peaked at around 270 ms in French-speaking 3–7-year-olds (Paquette et al., 2013). In several studies that used an oddball paradigm, deviants have elicited a second negative deflection in children, typically termed Late (Discriminative) Negativity (LDN; Čeponienė et al., 1998, 2003; Korpilahti et al., 2001; Kushnerenko et al., 2002). The late negative response to speech and non-speech contrasts has been shown to decrease with aging (Kraus et al., 1993; Bishop et al., 2011). On the basis of the reported results, we predicted that automatic discrimination between *der* and *den* will be indexed by an early and a late mismatch response.

So far, very few studies have investigated the auditory discrimination of naturally spoken functional words. For example, in the study by Endrass et al. (2004) who examined the modulations of MMN amplitude to word and non-word stimuli in adults, the German functional word *ab* [ap] elicited a greater MMN between 70 and 140 ms post-deviance than the pseudoword *ak* [ak]. These results corroborated with the findings of lexical enhancement both in adults (e.g., Diesch et al., 1998; Tavano et al., 2012) and in children (Korpilahti et al., 2001). The enhancement of the MMN amplitude in response to lexical items was explained by the existence of long-term memory traces for words, but not for pseudowords. Similarly, long-term memory traces were argued to have impact on the discrimination between phonemes of native and non-native language (Dehaene-Lambertz, 1997; Näätänen et al., 1997; Winkler et al., 1999; Sharma and Dorman, 2000). Experiments that used auditory training showed significant changes of the MMN to phonological contrasts as a function of training in adults (Kraus et al., 1995; Tremblay et al., 1997) and in full-term new-borns (Cheour et al., 2002a). Finally, MMN-indexed learning effects were demonstrated in 3- to 6-year-old children after 2 months of natural exposure to a foreign language (Cheour et al., 2002b).

Several studies with adult participants found that MMN can be modulated by the occurrence frequency of the word in a specific language (Shtyrov et al., 2011; Leminen et al., 2013; MacGregor and Shtyrov, 2013). For example, a study by Alexandrov et al. (2011) compared the mismatch response to Russian high-frequent *Mup* [m'ir] “peace, world” to low-frequent *Mop* [mor] “plague” in adults. The MMN response to the high-frequent word was 1.3 μV larger at Fz and peaked 56 ms earlier than the MMN to the low-frequent word. The frequency effect was argued to reflect the relative strength of the lexical representations that are associated with the frequent use of a given lexical item.

The determiner forms *der* and *den*, are polysemantic in the sense that they do not exclusively mark respective nominative and accusative masculine singular nouns. *Der* also marks dative feminine singular and genitive plural forms, while *den* is additionally used with dative plural nouns. Corpus-based analyses of the determiner forms *der* and *den* have revealed that, independently of the grammatical meaning, *der* occurs in adult speech more frequently than *den* (see Supplementary Material 1). Both in the written and spoken modalities, the relative occurrence frequency of the form *der* was at least double that of the relative frequency of the form *den*. The ratio of 2:1 was also observed in child-directed speech, as obtained from the analysis of spontaneous conversations between 13 3-year-old children (8 girls) and their parents, using the CHILDES data (MacWhinney, 2000). Analysis of children's speech confirmed this tendency. Based on the results of previous studies, occurrence frequency was predicted to modulate children's and adult's discriminative response. To test this hypothesis, we compared the mismatch response produced by high-frequent *der* to the mismatch response elicited by low-frequent *den* using the same-stimulus, or “identity MMN” (Pulvermüller et al., 2006). Identity MMN was calculated separately for each determiner form by subtracting the ERP response to this form in standard condition from the ERP response to this form in the deviant condition.

## Materials and methods

### Participants

Fifty-nine 3-year-old children participated in the MMN experiment. Informed parental consent was obtained for all children before the experiment, and children received a gift of their choice for participating in the study. The data of thirteen children were excluded from the ERP analysis due to a history of neurological disease (4 children), bilingual family environment (1 child), and the lack of at least 75% of artifact-free trials (9 children). To keep the sizes of age groups equal, a random sample of 34 children was chosen from the remaining 46 datasets for the present cross-developmental analyses (age range 3;1–3;11 years, mean age 3;6 years, standard deviation (*SD*) = 0;3 years, 21 girls).

Thirty-four adults (age range 21–35 years, mean age 27 years, *SD* = 3;8 years, 15 females) were recruited from the database of the Max Planck Institute for Cognitive and Brain Sciences, Leipzig. None of them reported any hearing or neurological deficits, and they were all monolingual German native speakers. Mean laterality quotient was 88% (range 50–100%, *SD* = 16%), as assessed by the German version of the Edinburgh Handedness Inventory (Oldfield, 1971). Written informed consent was obtained from all participants. The session also included an EEG experiment on processing syntactic complexity, and the order of experiments was counterbalanced. Adult participants were paid 21 Euros for their participation. The study was approved by the Research Ethics Committee of the University of Leipzig, Germany.

### Materials

**Figure 1:**
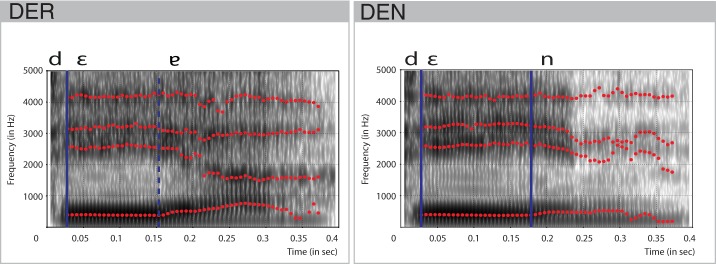
**Spectral characteristics of the stimulus items *den* and *der***. Formants F1, F2, F3, F4 are shown in red dotted lines. Blue vertical lines indicate transition points between the consonants and vowels. Dashed vertical blue line indicates the approximate transition point between two parts of the diphthong.

A trained, professional, female speaker recorded stimuli for *der* and *den* in a sound-isolated booth. They were matched for the intensity using root mean square amplitude and had a duration of 400 ms. Spectral characteristics of the items were analyzed using Praat (Boersma and David, 2015). The combination of the vocal [ε] and the uvular approximant [ʁ] at the end of *der* was realized as a phonetic diphthong [ε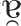] (cf. Kohler, 1995). Vocalization of the approximant started at 158 ms post-onset (Figure 1). The jaw-like opening constituted by the formants F2 and F3, slow growth of the formant F1, and perturbations in the formant F4 evidenced this. In *den*, the offset of the vowel [ε] laid approximately 30 ms behind this point. Hence, the point of physical deviance, that is, the point at which the difference between the forms could be detected, was defined at 158 ms. The stimuli were well matched for the fundamental frequency F0, as measured for the duration of the vowel (27–158 ms: 196 Hz) and for the transition period between vowel offset and consonant onset (158–371 ms: 165 Hz).

The 20-minute experiment consisted of four blocks with alternating standards. While *der* was presented as a standard in two non-successive blocks, *den* was established as a standard in the other two blocks. Block order was counterbalanced across participants. The stimulus items were presented with a fixed interstimulus interval of 500 ms from offset to onset of the next item in a pseudorandomized order. The first 10 items of each block were standards. In total, 126 standards were presented in each block with the occurrence probability of 83%.

### Procedure

EEG data was recorded at 129 electrode sites using Geodesic Sensor Nets (Electrical Geodesics, Inc., Eugene, OR, USA) with the operating impedance below 50 kΩ. The data was digitized online at a rate of 500 Hz. The electrode COM, placed next to the vertex, served as a common ground. The EEG recording with children was preceded by a warm-up session, during which an experimenter explained the procedure to the caregivers, played with the child, and introduced the sensor cap and recording cabin.

During the EEG recording, participants were seated in a comfortable chair in front of the VGA monitor (Sony, Tokyo, Japan) at a distance of 110 cm. A black-colored paper frame leaving a 29 × 22 cm window covered the monitor. During the experiment, silent cartoon films were shown. The stimuli were presented using Presentation (Neurobehavioral Systems, Inc., Albany, CA, USA) via Bowers and Wilkins loudspeakers (B&W Group Germany GmbH, Halle, Germany). Loudspeakers were located at approximately 140 cm in front of the participants. Small noiseless toys were allowed in the cabin if they did not cause excessive excitement. Participants' behavior was monitored via camera and microphones installed in the cabin.

### Data analysis

The data was down-sampled offline to 250 Hz, band-pass filtered between 0.3-20 Hz (Widmann, 2005) and re-referenced to linked mastoids (electrode sites E57 and E100). Algorithms exploiting Independent Component Analysis (ICA) were used to correct stereotyped artifacts such as vertical/horizontal eye movements and temporal muscle activity (Jung et al., 2000). Epochs time-locked to the stimulus onset were extracted. The length of the epoch was 1000 ms, including a 100-ms pre-stimulus baseline. Remaining artifact-contaminated epochs were automatically rejected if the amplitude of electrophysiological activity exceeded the absolute threshold of 150 μV and/or seven SDs of the mean probability distribution. The first 10 epochs were excluded from the analysis. Only standard items immediately preceding deviants were included into the individual ERPs, that is, the number of trials for standards and deviants was kept equal for all ERP comparisons. Three difference waves were calculated by subtracting (1) the response to the standard from the response to the deviant independently of the word, (2) the response to the standard *der* from the response to the deviant *der*, and (3) the response to the standard *den* from the response to the deviant *den*.

Nine regions of interest (ROIs) were defined in anterior-posterior and lateral planes: anterior-left, anterior-middle, anterior-right, central-left, central-middle, central-right, posterior-left, posterior-middle, posterior-right (for specific electrode sites included into each ROI, see Supplementary Material 2). Electrodes located at the edges of the cap were excluded from the analysis.

Statistical analyses were performed in two steps. Firstly, a potential time window for the MMN peak analysis was defined using separate ANOVAs with factors Stimulus type (Standard vs. Deviant), Anterior-posterior plane AP (Anterior vs. Central vs. Posterior), and Lateral plane LP (Left vs. Middle vs. Right) in 18 consecutive 50-ms windows between 0 and 900 ms. Corrections using the Greenhouse-Geisser method were applied if the assumption of sphericity was violated, as indicated by the Mauchly's test of sphericity.

In the second step, the local negative peaks were defined within MMN time windows at the electrode site E11 that corresponds to Fz in the nomenclature of the international 10–20 system. Peak amplitude and latencies were extracted automatically using the ERPLAB Measurement Tool (Lopez-Calderon and Luck, 2014). The developmental trajectory of the mismatch response was assessed using One-way ANOVAs for MMN peak latency and amplitude. Voltage topographies were computed for the peak amplitude of the difference wave in the resulting MMN window.

## Results

### All deviants

**Figure 2:**
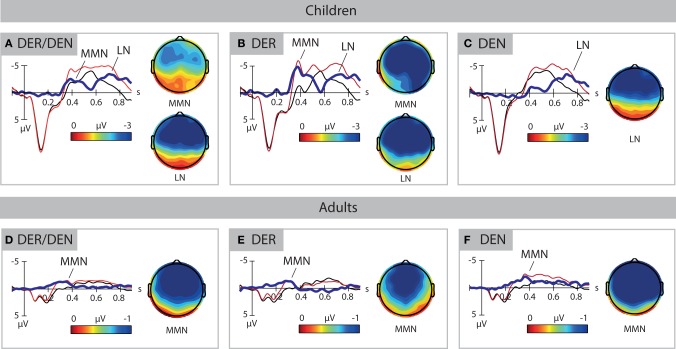
**ERP response to standards (in black) and deviants (in red)**. Voltage topographies are calculated for peak amplitudes of deviant-minus-standard difference wave (in blue), as reported in Table 1. The upper panels show the data of the children, the lower panels show the data of the adults. **(A,D)** shows the data for all deviants. **(B,E)** shows the data for the high-frequency deviant DER. **(C,F)** shows the data for the low-frequency deviant DEN. Negativity is plotted upwards. MMN, mismatch negativity; LN, late negativity.

Deviants elicited two clearly defined negativities in 3-year-old children, the MMN and the late negativity, shown in Figure 2A. ANOVAs performed in consecutive 50-ms time windows revealed the main effect of Stimulus type between 350 and 500 ms [*F*_(1, 33)_ = 12.224–23.560, *p* < 0.01] and between 600 and 900 ms [*F*_(1, 33)_ = 6.553–24.150, *p* < 0.05]. Analysis of the effect in the anterior-posterior plane between 700 and 900 ms indicated that the second negativity was distributed in anterior-central scalp areas. Thus, two time windows between 350 and 500 ms and 600 and 900 ms after word onset were chosen to define the peak of the mismatch response, reported in Table 1.

**Table 1:** **Mean peak amplitudes (in μV) and latencies (in ms) for the mismatch negativity (MMN) and late negativity (LN)**.

	**Children**				**Adults**	
	**MMN latency**	**MMN amplitude**	**LN latency**	**LN amplitude**	**MMN latency**	**MMN amplitude**
All	420 (46)	−3.85 (2.78)	742 (80)	−5.31 (3.41)	307 (52)	−2.29 (1.50)
DER	415 (62)	−7.08 (4.00)	745 (86)	−6.67 (4.01)	260 (69)	−2.94 (1.37)
DEN	–	–	718 (73)	−5.36 (4.19)	343 (52)	−3.06 (2.15)

Latencies are reported relative to the onset of the word.

Standard deviation (SD) is indicated in parentheses.

In adults, deviants elicited a classical MMN response that was significant between 200 and 400 ms [*F*_(1, 33)_ = 9.395–28.526, *p* < 0.01], shown in Figure 2D. Analysis of a Stimulus type × AP interaction revealed that the effect was present in anterior-central areas between 200 and 400 ms [*F*_(1, 33)_ = 7.569–27.236, *p* < 0.01] as well as in a posterior area between 250 and 350 ms [*F*_(1, 33)_ = 6.327–7.283, *p* < 0.05]. In time window 300–350 ms, an interaction Stimulus type × LP was found, but no hemisphere-specific distributions were observed. An interaction Stimulus type × AP × LP was also significant in this time window [*F*_(4, 132)_ = 2.775, *p* = 0.03]. The step-down analysis of the interaction showed that the effect was significant in all nine regions [*F*_(1, 33)_ = 9.467–28.827, *p* < 0.01]. Hence, the time period of 200–400 ms after word onset was chosen for quantification of the MMN in adults.

### High-frequency deviant *Der*

The mismatch negativities elicited by high and low frequency deviants had different patterns both in children and in adults. High-frequency deviant *der* elicited two negativities in 3-year-olds (Figure 2B). The main effect of Stimulus Type was observed between 300 and 550 ms [*F*_(1, 33)_ = 7.208–39.216, *p* < 0.05] and 600 and 900 ms [*F*_(1, 33)_ = 9.167–19.499, *p* < 0.01]. Interactions with factors AP and LP did not reveal any distribution-specific effects, apart of the time window 800–900 ms in which the difference between deviant and standard was only significant in anterior [*F*_(1, 33)_ = 10.772–11.472, *p* < 0.01] and central [*F*_(1, 33)_ = 9.891–13.559, *p* < 0.01] areas. Hence, time windows 300–550 ms and 600–900 ms after stimulus onset were chosen for the quantification of MMN to *der* in 3-year-olds.

In adults, the highly frequent deviant elicited an early negative deflection that was significant between 100 and 350 ms [*F*_(1, 33)_ = 7.327–19.923, *p* < 0.05], shown in Figure 2E. In time window 100–150 ms, an interaction of Stimulus type × AP × LP was observed [*F*_(3.08, 101.65)_ = 3.250, *p* = 0.024] that revealed region-specific distribution of the effect in anterior [*F*_(1, 33)_ = 4.995–9.345, *p* < 0.05] and central [*F*_(1, 33)_ = 6.863–14.709, *p* < 0.05] areas, as well as in the posterior-left region [*F*_(1, 33)_ = 6.311, *p* = 0.017]. Analysis of Stimulus type × LP interactions between 200 and 350 ms indicated a broad distribution of this effect. In time window 150–200 ms, the negativity was strongest in left and middle areas. On the basis of these results, time window 100–350 ms after stimulus onset was chosen for MMN quantification for *der* in adults. The mismatch response to *der* peaked significantly later in 3-year-olds than in adults [*F*_(1, 66)_ = 94.83, *p* < 0.001]. Its amplitude at the electrode site Fz differed significantly between age groups [*F*_(1, 66)_ = 32.69, *p* < 0.001].

### Low-frequency deviant *den*

Deviant determiner *den* elicited a late negativity between 600 and 900 ms after word onset in 3-year-olds (Figure 2C), as evidenced by the main effect of Stimulus type in these time windows [*F*_(1, 33)_ = 7.272–12.811, *p* < 0.01] and interactions with distributional factor AP (*Fs* = 3.817−11.340, *p* < 0.05). The follow-up analyses of the interactions confirmed an anterior-central focus of the effect [*F*_(1, 33)_ = 5.298–19.752, *p* < 0.05]. The late negativities for *der* and *den* did not differ significantly in children.

Determiner *den* elicited a sustained negative deflection in adults. It started at approximately 200 ms, peaked between 300 and 450 ms, and continued upon the following 400 ms after word onset (Figure 2F). Time-window analysis revealed the main effect of Stimulus type between 250 and 700 ms [*F*_(1, 33)_ = 6.761–22.903, *p* < 0.05], as well as a Stimulus type × AP interaction between 200 and 400 ms (*Fs* = 3.800–15.818, *p* < 0.05). Analyses of the interactions with the distributional factor indicated that the effect was distributed anterior-centrally between 200 and 400 ms [*F*_(1, 33)_ = 4.681–26.470, *p* < 0.05] and posteriorly between 300 and 400 ms [*F*_(1, 33)_ = 5.239–6.779, *p* < 0.05]. There were significant differences between MMN peak latencies [*F*_(1, 33)_ = 36.119, *p* < 0.001] for high and low frequent items in the adult group. Peak amplitudes did not show any significant effect.

## Discussion

The current MMN study was set out to investigate whether children at the age of 3 years are able to discriminate between the two forms of German definite determiner *der* and *den*. These forms unambiguously mark thematic roles of the verb arguments in a sentence when masculine singular nouns express them. There were two main findings in the current experiment. First, children showed a discriminative response to the determiner forms, namely an early (MMN) and a late (LN) negativity. Second, the highly frequent form *der* and the less frequent form *den* produced different ERP patterns. In children, the form *der* elicited an MMN and an LN, whereas the form *den* elicited only a late response. In adults, no LN response was observed, but the MNN elicited by *der* peaked 83 ms earlier than that elicited by *den*.

The first finding indicates that children at the age of three are able to pre-attentively discriminate between function words when presented beyond a sentential context. This is an important finding as it shows that the children's failure to correctly interpret sentences that crucially depend on these determiners is not due to a deficit in processing the auditory difference, but due to the grammatical function of these elements. The MMN observed in this study peaked at 420 ms after stimulus onset, that is, at 262 ms after the point of physical deviance. This latency fell into ranges reported by previous studies on speech and tone discrimination in 3-year-old children (e.g., Čeponienė et al., 2003; Glass et al., 2008; Putkinen et al., 2012; Paquette et al., 2013). In line with the studies that used oddball paradigms with young participants (e.g., Dehaene-Lambertz and Dehaene, 1994; Čeponienė et al., 1998; Kushnerenko et al., 2002), deviants elicited a late negativity with a peak at 584 ms after the point of deviation. The late negative response to speech and non-speech contrasts has been shown to decrease with age (Kraus et al., 1993; Cheour et al., 2001; Bishop et al., 2011). The presence of the sustained late negativity without a clearly defined peak in our adult data corroborates these findings. Taken together, the results of the present experiment challenge the account of high perceptual costs related to the acoustic processing of case markers as a barrier to sentence interpretation in early childhood.

The second finding suggests that both children's and adults' ability to discriminate between function words may be related to the psycho-linguistic features of the stimulus material such as the frequency of occurrence. Words with a higher frequency of use in a specific language were shown to elicit an enhanced MMN response (Pulvermüller et al., 2001; Shtyrov et al., 2011; Leminen et al., 2013; MacGregor and Shtyrov, 2013) with an earlier peak (Alexandrov et al., 2011). In our adult data, the more frequently used item *der* elicited an earlier MMN in comparison to the less frequently used item *den*. The less frequently used *den* did not elicit a significant MMN response in children. In accordance with previous findings, our results confirm that long-term memory representations of words have effect on the auditory discrimination both in children and adults. More familiar linguistic units appear to be easier to discriminate from others.

Support for this frequency-guided interpretation comes from the studies on the frequency effects in spoken word recognition that showed a faster reaction to frequently used items in adults (e.g., Marslen-Wilson, 1990). In ERP research, the frequency effect was indexed by latency shifts of the relevant ERP components such as P3b and early negativity (Polich and Donchin, 1988; Osterhout et al., 1997; King and Kutas, 1998). For example, Osterhout et al. (1997) recorded the EEG while participants were reading normal and scrambled prose. Regression analyses showed a negative correlation between the peak latency of the negative deflection that was elicited by words between 250 and 450 ms and the normative log word frequency. Electrophysiological correlates of the frequency effect in children have only been found at later latencies (>300 ms). For example, Berman and Friedman (1993) demonstrated that the processing of frequently used words was related to the decreased P3b latency in children at the age of 7-10 years. Taken together, these results indicate that more commonly used words elicit an earlier ERP response than less commonly used words both in adults and in children. This parallels with our findings of the earlier and a more mature MMN to the frequently used form *der* in our study.

One could argue that the morphological discrepancies between the difference waves obtained for *der* and *den* might be explained by factors other than word frequency, for example, by the change of acoustic parameters in standard-deviant sequence. Analysis of our naturally spoken stimulus items revealed that the stable vowel [ε] was 30 ms longer in *den* than in *der*. Thus, the difference wave for the determiner *der* was obtained from the sequence in which the length of the stable vowel increased between standard and deviant, whereas the difference wave for *den* involved duration increment. The latter co-occurred with the absence of the typical MMN. However, the increment of duration was reported to produce discriminative responses in children in previous research. For example, duration increment triggered two mismatch negativities in Finnish sleeping newborns that were presented with complex speech stimuli /asa/ and /assa/ (Kushnerenko et al., 2001). The first negativity peaked at about 150 ms and was also observed in one of the conditions in which infants were exposed to the consonant duration decrement of 160 ms. The second negativity peaked at about 350 ms and was evident for all duration deviances. Vowel duration increment also triggered an early (positive) discriminative response in 2-month-old German-acquiring infants (Friederici et al., 2002). These results were explained by the greater perceptual saliency of the long vowel deviants /ba:/ in contrast to the short deviants /ba/ that only elicited a late negativity. Also, preschool (mean age 5;4 years) and school Finnish children (mean age 9;3 years) in the study by Partanen et al. (2013) showed a statistically significant MMN to vowel duration increment of 80 ms in a word context for standard /tatata/ and deviant /tata:ta/. Altogether, these findings indicate that duration increment in speech stimuli triggers a mismatch response from an early age. Thus, acoustic discrepancies in vowel length can hardly explain the MMN asymmetry obtained in children. These effects, however, need to be clarified in further research.

In adults, duration increment of the stable vowel also co-occurred with a later MMN response to *den*, as compared to the duration decrement in response to *der*. However, peak latencies were reported to be similar for both decrement and increment MMN (e.g., Colin et al., 2009). In fact, independent of the deviance direction in standard-deviant sequence, the difference between two stimulus items is detected at the point of physical deviation (see also Peter et al., 2010). Therefore, the later peak of the MMN to deviant *den* in adults cannot be attributed to the vowel duration increment within the standard-deviant sequence.

Alternatively, the distinctive ERP patterns to *der* and *den* in our participants might be due to the contribution of purely acoustic features of the stimuli. Specifically, the absence of the significant MMN to *den* in children and a later MMN to *den* in adults, as compared to *der*, might be explained by differences in acoustic processing of two forms. While we are aware of the complexity of the phonological material in the present experiment, two aspects should be noted. First, the stimuli were matched in terms of the fundamental frequency, overall duration and intensity (for details, see section *Materials*). Second, an enhanced and earlier MMN during the processing of phonemic contrasts was primarily observed in experiments with “coronal” sounds deviating from the series of “dorsal” sounds than in case of dorsal deviants (Eulitz and Lahiri, 2004; Scharinger et al., 2010, 2012). This was not the case in the present study, in which the processing of the coronal [n] in *den* was associated with a smaller MMN in children and a later MMN in adults, as compared to dorsal [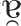] in *der*. Therefore, we prefer the frequency-based interpretation of MMN asymmetry in our participants to that related to acoustic features of two forms.

Our experiment demonstrated that while both children and adults are clearly able to differentiate between *der* and *den*, their discriminative abilities might be influenced by the distributional characteristics of the two forms. The less frequent form *den* elicited an immature response in 3-year-olds. Therefore, slower perceptibility of the accusative form, which is related to its low frequency in speech context, might contribute to the lacking reliance on accusative case marker *den* during the assignment of thematic roles in complex sentence. These results indicate that the impact of frequency of functional forms on general sentence interpretation in early childhood should not be underestimated.

### Conflict of interest statement

The authors declare that the research was conducted in the absence of any commercial or financial relationships that could be construed as a potential conflict of interest. The reviewer, George R. Mangun, and handling Editor, Tamara Swaab, declared their shared affiliation, and the handling Editor states that the process nevertheless met the standards of a fair and objective review.
