# Supplementary material for: Auditory Discrimination Between Function Words in Children and Adults: A Mismatch Negativity Study
Source: Front Psychol. 2015 Dec 22;6:1930. doi: 10.3389/fpsyg.2015.01930 (PMC4686640; doi:10.3389/fpsyg.2015.01930)
Supplement: Supplementary file 1 [file Table1.DOCX]

# Supplementary material 1

Table 1.1. Absolute and relative occurrence frequencies of the articles *der* and *den* in German corpora. Relative frequencies are normalized as per one million of tokens.

| **Corpus** | **Type of speech** | **Modality** | **Corpus size (in tokens)** | **Absolute frequency** | | **Relative frequency** | |
| --- | --- | --- | --- | --- | --- | --- | --- |
|  |  |  |  | ***der*** | ***den*** | ***der*** | ***den*** |
| Deutsches Textarchiv | A-to-A | written | 98964704 | 1436642 | 768636 | 14516.71 | 7767.77 |
| Kernkorpus 20 | A-to-A | written | 103432000 | 1909160 | 967205 | 18458.12 | 9351.12 |
| Kernkorpus 21 | A-to-A | written | 1547000 | 23234 | 14697 | 15018.75 | 9500.32 |
| Deutsches Referenzkorpus (DeReKo)* | A-to-A | written | 4278210573 | 142612969 | 47185166 | 333347.24 | 11029.18 |
| Gesprochene Sprache | A-to-A | spoken | 2500000 | 38317 | 17802 | 15326.8 | 7120.8 |
| Datenbank für Gesprochenes Deutsch (DGD)** | A-to-A | spoken | 7411613 | 127189 | 52519 | 17160.77 | 7086.04 |
| CHILDES*** | CH | spoken | 299134 | 6100 | 2427 | 20392.20 | 8113.42 |
| CHILDES*** | A-to-CH | spoken | 304276 | 4644 | 2174 | 15262.46 | 7144.83 |

*Note*: A-to-A, adult-to-adult speech; A-to-CH, adult-to-child speech; CH, child speech. * Only data from the subcorpus *Archiv der geschriebenen Sprache* was analyzed. ** Data from the subcorpus *Emigrantendeutsch in Israel* was excluded from the analysis. *** The analysis included 193 spontaneous conversations of 13 children (8 girls) at the age of 3;00-3;11 years.
