# Supplementary material for: Auditory Discrimination Between Function Words in Children and Adults: A Mismatch Negativity Study
Source: Front Psychol. 2015 Dec 22;6:1930. doi: 10.3389/fpsyg.2015.01930 (PMC4686640; doi:10.3389/fpsyg.2015.01930)
Supplement: Supplementary file 2 [file Table2.DOCX]

# Supplementary material 2

Table 2.1. Regions of interest defined for statistical analyses.

| ROI | Electrodes |
| --- | --- |
| Anterior-left (AL) | 32, 25, 26, 22 (Fp1), 23, 27, 33 (F7), 34, 28, 24 (F3), 20 |
| Anterior-middle (AM) | 21, 18, 15, 16, 11 (Fz), 12, 5, 4, 10, 14, 19 |
| Anterior-right (AR) | 9 (Fp2), 8, 3, 2, 1, 122 (F8), 123, 124 (F4), 116, 117, 118 |
| Central-left (CL) | 39, 40, 41, 42, 45 (T3), 46, 47, 35, 36 (C3), 37, 29, 30 |
| Central-middle (CM) | 13, 7, 129 (Cz), 31, 55, 54, 79, 80, 106, 112, 6 |
| Central-right (CR) | 108 (T4), 109, 110, 111, 102, 103, 104 (C4), 105, 115, 87, 93, 98 |
| Posterior-left (PL) | 50, 51, 52 (P3), 53, 58 (T5), 59, 60, 64, 65, 66, 70 (O1) |
| Posterior-middle (PM) | 61, 62 (Pz), 78, 67, 72, 77, 71, 76, 75 (Oz), 74, 82 |
| Posterior-right (PR) | 83 (O2), 84, 85, 86, 90, 91, 92 (P4), 95, 96 (T6), 101, 97 |

*Note*. Labels of the standard 10-20 system are indicated in parentheses.


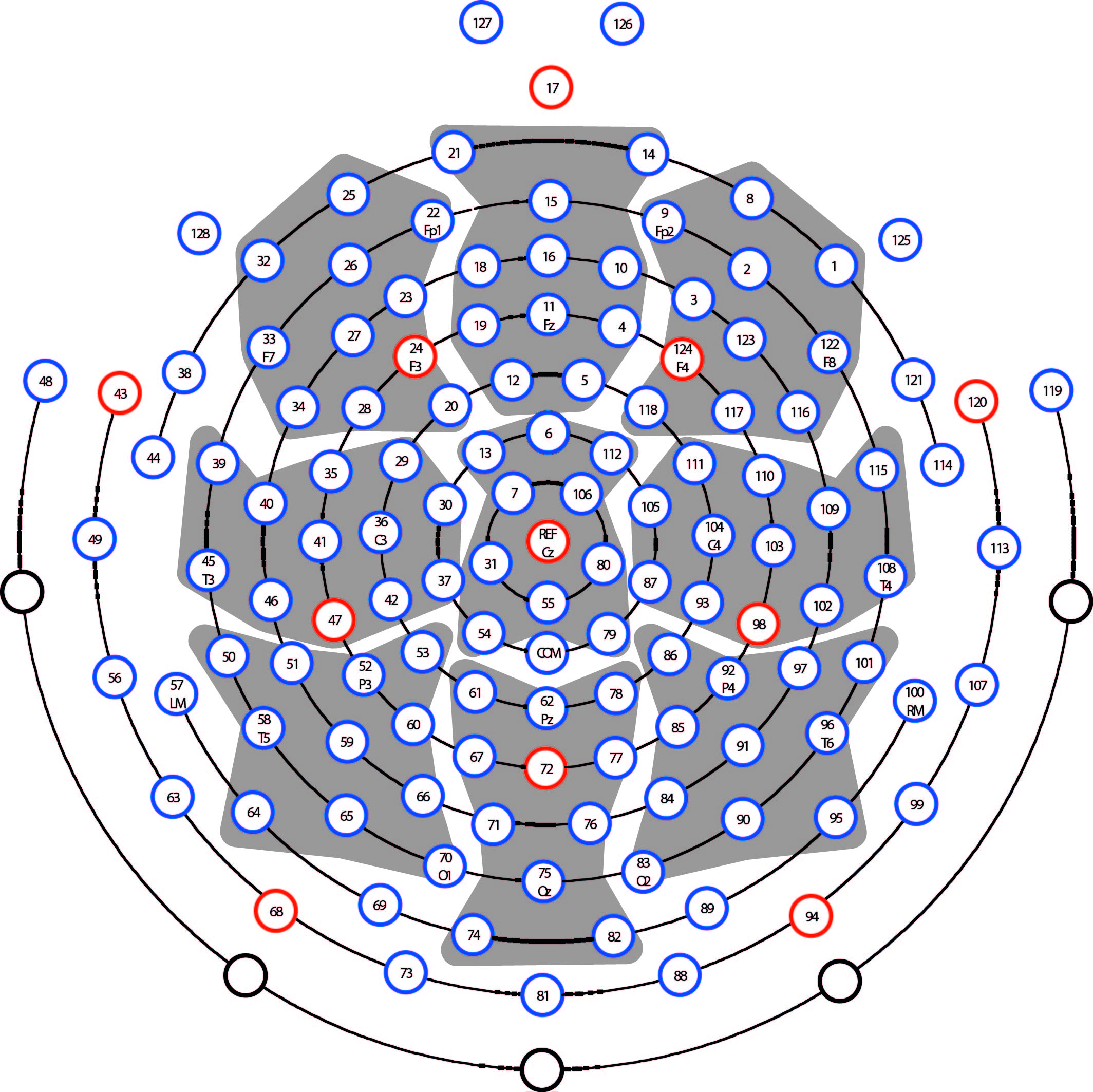


Figure 2.1. 129-channel map showing nine regions of interest.
